# High Serum Cyclophilin C levels as a risk factor marker for Coronary Artery Disease

**DOI:** 10.1038/s41598-019-46988-x

**Published:** 2019-07-22

**Authors:** Amparo Alfonso, Jeremías Bayón, Sandra Gegunde, Eva Alonso, Rebeca Alvariño, Melisa Santás-Álvarez, Ana Testa-Fernández, Ramón Rios-Vázquez, Carlos González-Juanatey, Luis M. Botana

**Affiliations:** 1 Pharmacology Department, Facultad de Veterinaria, Universidad de Santiago de Compostela, 27002 Lugo, Spain; 2 Cardiology Department, Hospital Universitario Lucus Augusti, 27003 Lugo, Spain

**Keywords:** Diagnostic markers, Cardiovascular biology

## Abstract

Cyclophilins (Cyps) are ubiquitous proteins that belong to the immunophilins family consistently associated with inflammatory and cardiovascular diseases. While levels of CypA have been extensively studied, less data are available for other Cyps. The purpose of this case-control study was to determine the relationship of Cyps (A, B, C and D) with coronary artery disease (CAD) and eight inflammation markers. Serum levels of Cyps, interleukins and metalloproteinases were measured in serum collected from 84 subjects. Participants were divided into two sub-groups based on CAD diagnosis: 40 CAD patients and 44 control volunteers. Serum levels of CypA, CypB and CypC, IL-1β and IL-6 were significantly higher in CAD patients. Bivariate correlation analysis revealed a significant positive correlation between Cyps and several blood and biochemical parameters. When the ability of Cyps levels for CAD diagnosis was evaluated, higher sensitivity and selectivity values were obtained with CypC (c-statistic 0.891, p < 0.001) indicating that it is a good marker of CAD disease, while less conclusive results were obtained with CypA (c-statistic 0.748, p < 0.001) and CypB (c-statistic 0.655, p < 0.014). In addition, significant correlations of traditional CAD risk factors and CypC were observed. In summary, high levels of CypC are a risk factor for CAD and therefore it can be proposed as a new biomarker for this disease.

## Introduction

Coronary artery disease (CAD) is an ischemic heart disease of coronary arteries mainly caused by atherosclerosis. The risk of CAD is increased in patients with traditional cardiovascular risk factors such as diabetes mellitus, age, tobacco, high blood pressure, chronic kidney disease, high lipid levels, overweight and obesity^1–4^. In addition, there is a higher incidence in mortality when CAD is associated to inflammatory and autoimmune diseases, being systemic inflammation a key factor in the development of this disease^5^. Besides, there is a general agreement of the fact that atherosclerosis is an inflammatory disease, and that inflammation plays an important role in the atherogenic process. Thus, inflammation contributes to all stages of atherosclerosis, from plaque formation to instability and eventual plaque rupture^5–8^.

Immunophilins are a family of peptidyl-prolyl cis/trans isomerases (PPIases) consistently involved in cardiovascular diseases^9^. PPIases include three protein subfamilies, Cyclophilins (Cyps), FK506-binding proteins (FKBP) and parvulins. Some of these proteins, namely CypA, CypB, CypD or FKBP51 have been previously associated with CAD^10–13^. Cyps are a highly conserved family of ubiquitous enzymes with at least 17 types. They catalyze cis-trans isomerization of protein peptidyl-prolyl bonds and modulate protein folding and assembly. CypA, CypB and CypC are secreted to the extracellular space while CypD is usually localized in the mitochondria^9^. CypA is secreted to the extracellular media after inflammatory stimuli and both intra and extracellular CypA are involved in several cardiovascular diseases^9,14^. Intracellular CypA plays a role in NOX enzyme translocation, Janus and ERK1/2 activation, Reactive Oxygen Species (ROS) production and oxidative stress^9^. CypB is expressed at lower levels than CypA and has an important role in redox hemostasis and inflammation with different effects depending on intracellular or extracellular localization and in the vascular function^9,15^. Both, CypA and CypB, are ligands of the Extracellular Matrix Metalloproteinase Inducer (EMMPRIN) (CD147) receptor^16^. These proteins are constitutively secreted through vesicles by endothelial and vascular smooth muscle cells (VSMC), fibroblasts, macrophages and platelets in response to oxidative stress. Moreover, CypA acts as a chemoattractant along with cytokines in inflammatory cells and CypB was also described as a chemotaxis inducer of inflammatory cells into damage tissues^9^. CypC is localized in the kidney and modulates macrophage activation, endotoxin signaling, and metalloproteinase-13 expression and it is also involved in macrophages activation and redox homeostasis of endoplasmic reticulum together with CypB^15,17^. The cyclophilin C-associated protein is the endogenous ligand for CypC that regulates the response to endotoxins and macrophages activation^17^. CypD plays a role in the opening of mitochondrial permeability transition pore (mPTP), and has been linked to apoptosis, Alzheimer’s disease and heart failure^12^.

While the role, mode of action and localization of CypA in cardiovascular diseases is well documented and it has been proposed as a biomarker for CAD, less data are available about other Cyps, in terms of cellular targets or extracellular levels, and the relationship of those when other cardiovascular risk factors are present^9,14,16,18^. Besides, as it was mentioned, the relationship of Cyps and inflammation has been extensively discussed. Extracellular Cyps levels are considered as an inflammatory response to injury since they are increased in inflammatory tissues and body fluids in many acute and chronic inflammatory processes^19,20^. In addition, Cyps are related to redox homeostasis, and redox mediated signaling has an important role in inflammation, atherosclerosis and CAD^15,21,22^. Taking into account the multiple cross-talks of these enzymes, the aim of this paper was to explore the levels of CypA, B, C and D and several inflammatory markers in CAD patients in order to better understand and predict this disease.

## Results

**Figure 1:**
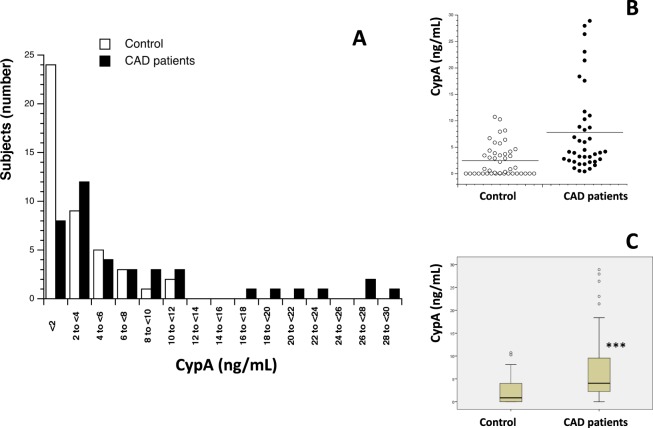
Serum Cyclophilin A (CypA) levels in control subjects (n = 44) and in patients (n = 40) with coronary artery disease (CAD). (**A**) Frequency plot: Data represented as CypA levels (X-axis) *versus* number of subjects (n = 84) (Y-axis). (**B**) Data are shown as dots of CypA levels in control subjects (control, n = 44) and CAD patients (n = 40). (**C**) Data are shown as box-and-whisker plots of CypA levels in control subjects (n = 44) and CAD patients (n = 40). CypA was significantly elevated (***P < 0.001, Mann-Whitney U test) in CAD patients compared with controls.

**Figure 2:**
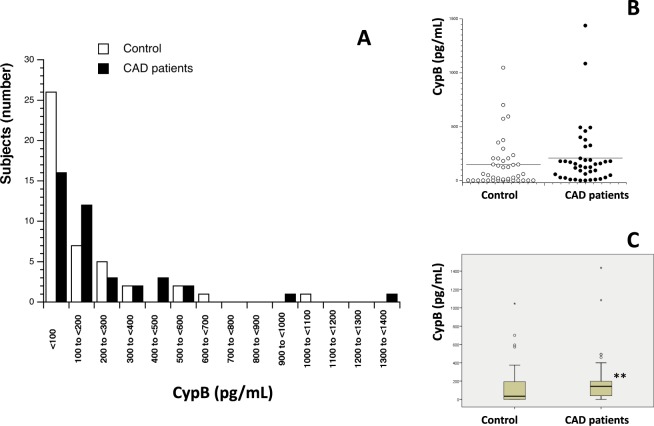
Serum Cyclophilin B (CypB) levels in control subjects (n = 44) and in patients (n = 40) with coronary artery disease (CAD). (**A**) Frequency plot: Data represented as CypB levels (X-axis) *versus* number of subjects (n = 84) (Y-axis). (**B**) Data are shown as dots of CypB levels in control subjects (control, n = 44) and CAD patients (n = 40). (**C**) Data are shown as box-and-whisker plots of CypB levels in control subjects (n = 44) and CAD patients (n = 40). CypB was significantly elevated (**P < 0.01, Mann-Whitney U test) in CAD patients compared with controls.

**Figure 3:**
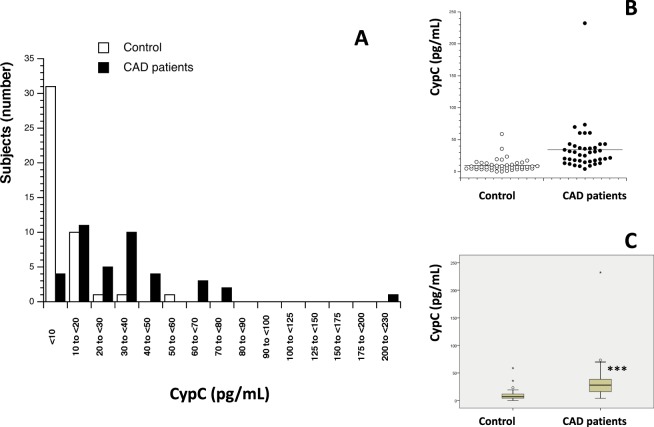
Serum Cyclophilin C (CypC) levels in control subjects (n = 44) and in patients (n = 40) with coronary artery disease (CAD). (**A**) Frequency plot: Data represented as CypC levels (X-axis) *versus* number of subjects (n = 84) (Y-axis). (**B**) Data are shown as dots of CypC levels in control subjects (n = 44) and CAD patients (n = 40). (**C**) Data are shown as box-and-whisker plots of CypC levels in control subjects (n = 44) and CAD patients (n=40). CypC was significantly elevated (***P < 0.001, Mann-Whitney U test) in CAD patients compared with controls.

**Figure 4:**
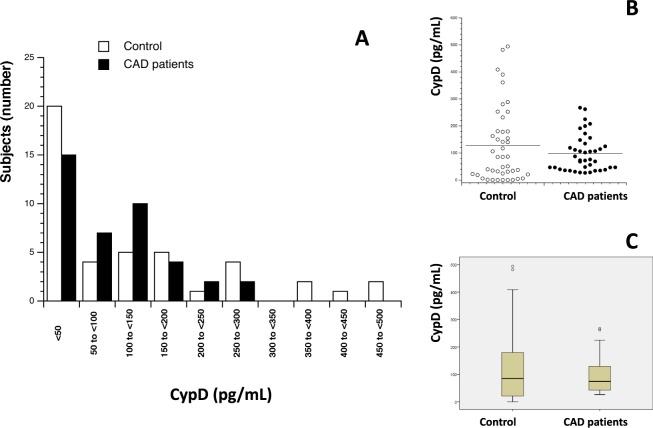
Serum Cyclophilin D (CypD) levels in control subjects (n = 44) and in patients (n = 40) with coronary artery disease (CAD). (**A**) Frequency plot: Data represented as CypD levels (X-axis) *versus* number of subjects (n = 84) (Y-axis). (**B**) Data are shown as dots of CypD levels in control subjects (n = 44) and CAD patients (n = 40). (**C**) Data are shown as box-and-whisker plots of CypD levels in control subjects (n = 44) and CAD patients (n = 40).

84 participants (mean age 54.3 ± 1.58, range 26–85 years, 66.7% male) were enrolled in the present study and divided into two sub-groups based on CAD diagnosis: 40 CAD patients (35 men and 5 women) and 44 control volunteers (CAD free) (22 men and 22 women). The same measurements were done in both populations to compare results. First, serum levels of Cyp A, B, C and D were measured (Table 1). Figures 1–4 show the distribution of patients with and without CAD by serum levels of Cyps and the distribution of Cyp levels among the groups under study (CAD patients and control volunteers). As it was expected, the levels of CypA, Fig. 1, were significantly increased in CAD patients, 7.80 ng/mL ± 1.02 compared with control subjects, 2.44 ng/mL ± 0.46 (P < 0.001). When CypB levels were compared, as shown in Fig. 2, the values in CAD patients were significantly higher than in control participants, 208.83 pg/mL ± 36.18 and 148.69 pg/mL ± 35.34 (P < 0.05), respectively. In the case of CypC, Fig. 3, the levels in CAD patients were significantly higher, 34.28 pg/mL ± 5.77 *versus* 9.60 pg/mL ± 1.52 (P < 0.001). However, when CypD levels were measured, Fig. 4, no significant differences were observed, 97.68 pg/mL ± 10.69 in CAD patients *versus* 127.33 pg/mL ± 20.84 in control subjects. Furthermore, levels of eight inflammation markers, IFNϒ, IL-1β, IL-2, IL-6, IL-8, TNFα, MMP-2 and MMP-9 were also analyzed to understand their relationship with immunophilins production and CAD. As Table 1 shows, no significant modifications in the levels of these parameters were observed when CAD patients and control participants were compared, with three exceptions, IL-1β, IL-6 and MMP-2. IL-6 levels were significantly increased in CAD patients, 8.08 pg/mL ± 1.59, with respect to control subjects, 3.82 ± 0.30, (P < 0.05). Also, IL-1β levels were significantly increased in CAD patients, 3.73 pg/mL ± 0.36, *versus* 2.71 pg/mL ± 0.17 for control subjects (P < 0.05). On the contrary, MMP-2 levels were significantly decreased, from 120.9 ng/mL ± 5.11 in control subjects to 95.43 ng/mL ± 5.69 in CAD patients (P < 0.001).

**Table 1:** Human serum levels of Cyclophilins and inflammatory markers in control subjects and coronary artery disease patients.

	Control subjects (n = 44)	CAD patients (n = 40)	*P* Value
CypA (ng/mL)	2.44 ± 0.46	7.80 ± 1.30	<0.001
CypB (pg/mL)	138.55 ± 33.39	208.83 ± 44.76	0.014
CypC (pg/mL)	9.60 ± 1.52	34.28 ± 5.77	<0.001
CypD (pg/mL)	127.33 ± 20.84	97.68 ± 10.69	0.687
IFNϒ (pg/mL)	34.26 ± 2.89	27.43 ± 2.65	0.073
IL-1β (pg/mL)	2.72 ± 0.17	3.73 ± 0.36	0.016
IL-2 (pg/mL)	4.32 ± 0.45	4.97 ± 0.51	0.543
IL-6 (pg/mL)	3.98 ± 0.33	8.08 ± 1.59	0.028
IL-8 (pg/mL)	8.01 ± 0.64	7.02 ± 0.75	0.131
TNFα (pg/mL)	10.34 ± 0.47	9.27 ± 0.64	0.123
MMP-2 (ng/mL)	121.53 ± 5.03	95.43 ± 5.69	<0.001
MMP-9 (ng/mL)	256.85 ± 45.81	221.37 ± 20.44	0.087

Data are shown as average ± SEM. Significance (p < 0.05) comparing CAD patients and control subjects. Coronary artery disease (CAD), Cyclophilin A (CypA), Cyclophilin B (CypB), Cyclophilin C (CypC), Cyclophilin D (CypD). Interferon ϒ (IFNϒ), interleukin (IL), tumor necrosis factor α (TNFα), metalloproteinase (MMP). Student’s T test or Mann-Whitney test were used to perform statistical analysis. Significant differences p < 0.05.

At the same time, traditional baseline characteristics as well as laboratory data of participants were collected and are shown in Table 2. Results were divided in two populations. As Table 2 shows, a higher percentage of patients with CAD were smoker or ex-smoker, with history of cardiac failure and some of them had peripheral arterial disease. In addition, HTA, Diabetes Mellitus Type 1 and Type 2 or DL diagnosis were also prevalent in CAD patients. Besides, clinical parameters as total cholesterol, LDL and HDL cholesterol, triglycerides (TG), C-reactive protein (CRP) and alanine aminotransferase (ALT) were also measured. While TG, CRP and ALT were significantly increased in CAD patients, cholesterol values were significantly decreased, according to statins treatment in this cardiovascular high-risk populations. In the case of blood cell count, white blood cells (WBC), neutrophils, monocytes and hemoglobin were significantly increased in CAD patients as well as glucose levels, while lymphocytes and platelets were not modified. Significantly more CAD patients were under treatment with statins, antiplatelets/anticoagulant, antihypertensives and hypoglycemics.

**Table 2:** Baseline clinical characteristics in control subjects and Coronary Artery Disease (CAD) patients.

	Control subjects (n = 44)	CAD patients (n = 40)	*P* Value
Male (%)	50%	87.5%	0.001
Smoker	2.3%	37.5%	<0.001
Ex-smoker	15.9%	27.5%	<0.001
Family History of CAD	18.2%	17.5%	0.900
CF History	0.0%	15%	0.009
Peripheral arterial disease	0.0%	7.5%	0.064
**Medical history**			
Hypertension (HTA)	6.8%	47.5%	<0.001
Dibetes Mellitus Type 1	0.0%	2.5%	<0.05
Dibetes Mellitus Type 2	2.3%	17.5%	<0.05
Dyslipidemia (DL)	13.6%	67.5%	<0.001
Total Cholesterol (mg/dL)	202.57 ± 1.54	177.05 ± 6.74	0.004
LDL (mg/dL)	122.03 ± 3.90	108.00 ± 5.54	0.071
HDL (mg/dL)	58.00 ± 2.55	36.53 ± 1.29	<0.001
TG (mg/dL)	106.89 ± 6.84	164.00 ± 11.31	<0.001
CRP* (mg/dL)	0.49 ± 0.14	43.45 ± 15.10	<0.001
ALT (U/L)	22.06 ± 2.38	33.32 ± 3.74	0.003
WBC (number/µL)	6632.35 ± 320.45	10383.50 ± 748.25	<0.001
Lymphocytes (number/µL)	2139.39 ± 113,98	1982.50 ± 184.63	0.119
Neutrophils (number/µL)	3757.58 ± 293.36	7462.50 ± 670.15	<0.001
Monocytes (number/µL)	548.48 ± 23.08	730.00 ± 49.90	0.01
Hemoglobine (pg)	13.91 ± 0.23	14.65 ± 0.24	0.028
Platelet (number/µL)	230205.88 ± 8777.89	221925.00 ± 12331.08	0.648
Glucose (mg/dL)	90.23 ± 5.48	110.80 ± 3.16	0.001
**Medications**			
Statins	11.4%	42.5%	0.003
Antiaplatelets/anticoagulant	0.0%	22.5%	0.001
Antihypertensives	9.1%	52.5%	<0.001
Hypoglycemics	2.4%	15.0%	0.05
**Angiographyc findings**			
1-vessel disease	0.0%	47.5%	N/A
2-vessel disease	0.0%	30.0%	N/A
3-vessel disease	0.0%	22.5%	N/A
Total Revasc	0.0%	70.0%	N/A

Data are shown as average ± SEM. Significance (p < 0.05) comparing CAD patients and control subjects. Cardiac failure (CF), low density lipoprotein (LDL), high-density lipoprotein (HDL), triglycerides (TG), C-reactive protein (CRP), alanine aminotransferase (ALT), white blood cells (WBC). *N = 30. Student’s T test or Mann-Whitney test were used to perform statistical analysis. Categorical variables were compared using *Χ*^2^ test. Significant differences p < 0.05.

**Figure 5:**
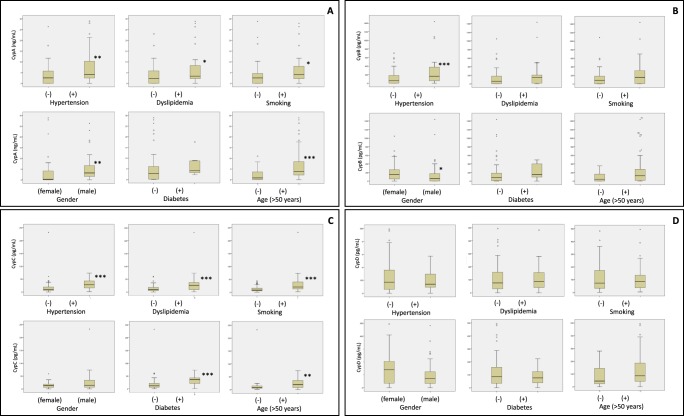
Cyclophilin levels and cardiovascular risk factors (hypertension, dyslipidemia, smoking, gender, diabetes, age > 50). (**A**) Cyclophilin A (CypA) levels were significantly increased by the presence of hypertension, dyslipidemia, smoking, age of 50 years or older and in men but not by diabetes. (**B**) Cyclophilin B (CypB) levels were significantly increased by the presence of hypertension and in men but not by dyslipidemia, smoking, diabetes or age of 50 years or older. (**C**) Cyclophilin C (CypC) levels were significantly increased by the presence of hypertension, dyslipidemia, smoking, diabetes and age of 50 years or older but not by gender difference. (**D**) Cyclophilin D (CypD) levels were not significantly increased by the presence of cardiovascular risk factors. Mann-Whitney U Test was used to check statistic significances, N = 84. (***P < 0.001, **P < 0.01, *P < 0.05).

Bivariate correlation analysis of Cyps and clinical parameters revealed, Table 3, a significant positive correlation between CypC levels and the levels of CypA (p < 0.002), CypB (p < 0.001) and CypD (p < 0.01). In addition, CypC was positively associated to IL-6, WBC, neutrophils and glucose. Furthermore, CypC was negatively associated with MMP-2 and HDL levels. In the case of CypA, there is a significant positive correlation between the levels of this immunophilin and IL-1β, TG, ALT, neutrophils and glucose levels, while a negative correlation is observed with IL-8, TNFα, MMP-2 and HDL. CypB levels were positively associated with CypD, IL-6 and MMP-9 measures and negatively with hemoglobin levels. Finally, CypD serum levels were only positively associated with MMP-9 and platelet. No correlations of Cyps levels with IFNϒ, IL-2, total cholesterol, LDL, lymphocytes and monocytes were observed. Besides CypA levels were significantly (p < 0.05) elevated in subjects with traditional cardiovascular risk factors such as hypertension, dyslipidemia, smoking, gender (male) and age > 50 years, Fig. 5A. In the case of CypB, Fig. 5B, the levels were significantly elevated in subjects with hypertension (p < 0.01) and gender (male) (p < 0.05). Interesting CypC was significantly elevated (p < 0.002) by the presence of all cardiovascular risk factors with the exception of gender, Fig. 5C. Finally, CypD levels were not related to the risk factors, Fig. 5D.

**Table 3:** Correlations between cyclophilins, inflammation markers and clinical parameters.

	CypA	CypB	CypC	CypD
CypA			0.339 (p < 0.005)	
CypB			0.535 (p < 0.001)	0.389 (p < 0.001)
CypC	0.339 (p < 0.002)	0.535 (p < 0.001)		0.284 (p < 0.01)
CypD		0.389 (p < 0.001)	0.284 (p < 0.01)	
IL-1β	0.304 (p < 0.01)			
IL-6		0.257 (p < 0.01)	0.302 (p < 0.005)	
IL-8	−0.300 (p < 0.005)			
TNFα	−0.298 (p < 0.005)			
MMP-2	−0.270 (p < 0.01)		−0.215 (p < 0.05)	
MMP-9		0.304 (p < 0.005)		0.378 (p < 0.001)
HDL	−0.294 (p < 0.01)		−0.466 (p < 0.001)	
TG	0.266 (p < 0.05)			
CRP	0.409 (p < 0.05)		0.577 (p < 0.005)	
ALT	0.332 (p < 0.005)			
WBC	0.219 (p < 0.05)		0.310 (p < 0.005)	
Neutrophils	0.296 (p < 0.01)		0.379 (p < 0.001)	
Hemoglobin		−0.238 (p < 0.05)		
Platelet				0.262 (p < 0.05)
Glucose	0.363 (p < 0.001)		0.520 (p < 0.001)	

Bivariate analysis, N = 84. Spearman coefficient and significance (p < 0.05). Significant differences p < 0.05. Abbreviations as in Tables 1 and 2.

**Figure 6:**
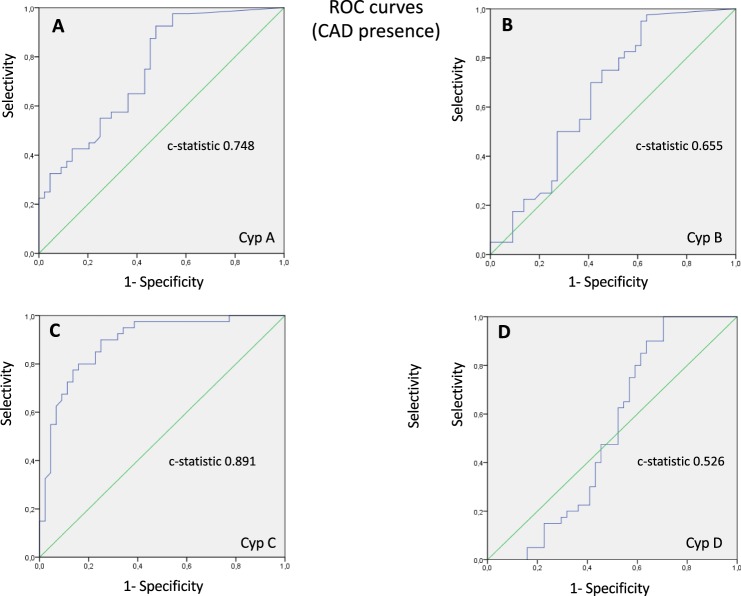
Receiver-operating-characteristic curves (ROC) describing the diagnostic performance of cyclophilins to identify coronary artery disease (CAD) > 50% as compared with the control subjects. (**A**) Cyclophilin A (CypA), the AUC (c-statistic) was 0.748 (95% confidence interval (CI), 0.654–0.851, p < 0.001). (**B**) Cyclophilin B (CypB), the AUC (c-statistic) was 0.655 (95% CI, 0.538–0.773, p = 0.014). (**C**) Cyclophilin C (CypC), the AUC (c-statistic) was 0.891 (95% CI, 0.821–0.961, p < 0.001), Specificity (88.6%), Sensitivity (70%), positive predictive value (VPP) (84.8%) and negative predictive value (VPN) (76%). (**D**) Cyclophilin D (CypD) the AUC (c-statistic) was 0.526 (95% CI, 0.396–0.655, p = 0.687). N = 84.

Then, to evaluate the sensitivity and selectivity of Cyps values for the diagnosis of CAD, ROC curves were calculated on the basis of immunophilin levels, using CAD presence or absence as state variable or specific condition. As Fig. 6 shows, higher sensitivity and selectivity values were obtained with CypC. The AUC (c-statistic) calculated was 0.891 showing a significant value (p < 0.001) for CypC as predictor of CAD. For CypA and CypB, AUC were also significant, 0.748 and 0.655 respectively while CypD values are not useful to predict CAD. Therefore, it can be concluded that CypC is a good marker, CypA is fair as marker while CypB is a poor marker. From ROC curves, cut-off points for CAD presence were obtained as a compromise between sensitivity and selectivity. A cut-off for CypA > 8.2 ng/mL provides a specificity of 95.5%, a sensitivity of 32.5%, a positive predictive value (VPP) of 86.7%, a negative predictive value (VPN) of 60.9% and statistic power (P) of 65.5%. A cut-off for CypB > 204 pg/mL provides a specificity of 77.3%, a sensitivity of 25%, a VPP of 50%, a VPN of 53.1% and P of 52.4%. In the case of CypC, a cut-off > 17.5 pg/mL provides a specificity of 88.6%, a sensitivity of 70%, a VPP of 84.8%, a VPN of 76.5% and P of 79.8%. These values were used to evaluate the association between serum Cyps levels and traditional risk factors (gender, age, smoke, dyslipidemia and HTA). Each of the known risk factors and Cyps were combined in a single logistic-regression model (Table 4). When CypB > 204 pg/mL and CAD correlations were studied (univariable analysis) no significant differences were observed (data not shown), therefore this protein was not used for multivariable analysis. However, as Table 4 shows, either traditional risk factors and CypA and C are highly related with disease status (OR values between 7 and 18.2 and p > 0.005), with a higher relationship in the case of CypC. When multivariable analyses were performed with traditional risk factors, OR values were decreased while CI increased, compared to univariable analysis, but still significant. When CypA was added to risk factors, both age and CypA lost the correlation with CAD, while the rest of risk factors remained highly related with the disease. The inclusion of CypC in multivariable analysis, instead of CypA, resulted in a significant improvement of the overall performance of the logistic regression model (Cox-Snell R^2^ from 0.54 to 0.596), although CI were also increased, and age and smoking condition lost the correlation with CAD. Finally, when both, CypA and CypC, were included in the logistic regression model, R^2^ was improved to 0.605 as well as the CI. In summary, additionally to traditional risk CAD factors, levels of CypC over 17.5 pg/mL or of CypA over 8.2 ng/mL increase the risk of CAD. Therefore, CypA serum levels but especially the level of CypC can be useful biomarkers for CAD.

**Table 4:** Correlated Risk factors for coronary artery disease.

	Univariable analysis			Multivariable analysis (1)			Multivariable analysis (2)			Multivariable analysis (3)			Multivariable analysis (4)		
	OR	95% CI	P value	OR	95% CI	P value	OR	95% CI	P value	OR	95% CI	P value	OR	95% CI	P value
Age > 50	8.18	2.8–23.5	<0.001	5.927	1.2–29.2	0.029	4.208	0.7–22.9	0.097	3.733	0.9–23	0.156	2.455	0.4–15	0.33
Gender (male)	7	2.3–21.1	0.001	10.835	1.2–80.4	0.020	14.064	1.5–130.4	0.02	27.026	2.3–316.5	0.009	18.725	1.8–194.7	0.014
Smoking	8.357	3–22.8	<0.001	14.731	2.8–76.1	0.001	13.499	2.4–74.18	0.003	5.872	0.9–36.4	0.057	4.757	0.8–27.4	0.081
HTA	12.36	3.2–46.5	<0.001	12.714	1.4–109.1	0.020	12.709	1.4–113.2	0.023	22.227	1.8–265.7	0.014	8.989	1.1–70	0.04
DL	13.15	4.4–38.9	<0.001	6.065	1.24–29.6	0.026	5.594	1–30.3	0.046	7.113	1.2–42.7	0.032	5.18	0.9–29.4	0.064
Cyp A > 8.2 ng/mL	10.11	2.1–48.3	0.004				5.177	0.7–38.2	0.107				4.504	0.5–36	0.156
Cyp C > 17.5 pg/mL	18.2	5.7–57.5	<0.001							23.002	2.5–209.8	0.005	18.46	2.2–152.6	0.007

Analysis was performed on 7 variables: (1) Traditional risk factors (age > 50, gender, smoking, HTA and DL), % of cases 88.0, Cox and Snell R^2^: 0.538, Nagelkerke R^2^: 0.717. (2) Traditional risk factors + CypA, % of cases 86.7, Cox and Snell R^2^: 0.554, Nagelkerke R^2^: 0.739. (3) Traditional risk factors + CypC, % of cases 88.0, Cox and Snell R^2^: 0.596, Nagelkerke R^2^: 0.796. (4) Traditional risk factors + CypA + CypC, % of cases 90.4, Cox and Snell R^2^: 0.605, Nagelkerke R^2^: 0.807. Significant differences p < 0.05.

Abbreviations as in Tables 1 and 2.

## Discussion

Cyps play a key role in protein folding, repairing damaged proteins, modulation of the immune system, and other biological functions, although the role of most of the components of this family is not well known^23,24^. In addition to physiological functions, these proteins have been related with many other processes. In this regard, Cyps have been associated to cancer, aging, diabetes mellitus, chronic kidney diseases and neurodegeneration among others. Nevertheless, they seem to play a especial, and critical role in cardiovascular diseases^9,12^. In fact, CypA plasma levels have been previously proposed as biomarkers for coronary artery disease having also prognostic impact^18,21^. However, the role of the other Cyps and their relationship with CypA is less known. In the present study, we describe the serum levels of 4 Cyps and 8 inflammation markers in patients with acute CAD in comparison to healthy individuals, in order to clarify their relationship and to know if the less-studied immunophilins have a role in this disease.

CypA is released to the extracellular media in response to inflammatory stimuli and it is a critical regulator of endothelial cell dysfunction, vascular smooth muscle cells, fibroblast proliferation and cardiac hypertrophy^9,12^. In addition, CypA is involved in atherosclerosis, in oxidative stress, in nitric oxide generation and probably in blood pressure regulation^9,12^. We observed that CypA levels were significantly increased in CAD patients, as it was previously described^18,21,25^. In addition, the levels of CypA were correlated with several clinical parameters and with several inflammation markers as IL-1β, IL-8, TNFα, MMP-2, as well as with CRP, a known clinical marker of vascular inflammation. The correlation of CypA and CRP was before described and the role of both to predict the risk of coronary artery disease, or as markers of proinflammatory status in patients with diabetes was hypothesized^11,18,21,25^. Extracellular CypA has been involved in the production of IL-8, IL-1β, TNFα and MMP-9, while MMP-2 was not affected^26,27^. From our results, including ROC values, the role of CypA as CAD biomarker is reinforced although no significant correlation with traditional CAD risk factor was observed, probably due to the limited population used.

CypB is also abundant in all tissues but at lower levels than CypA. It has a crucial role against ROS and pro-inflammatory stimuli ant to induce chemotaxis of inflammatory cells. In addition, CypB levels were associated with the prevalence of metabolic syndrome and it is also related to hypertension, ROS effects in vascular function and heart failure^9,13,28^. In the present study, CypB levels were significantly increased in CAD patients and this increase was associated with IL-1β, IL-6, and MMP-9. However, CypB for CAD diagnosis, with a c-statistic 0.655 seems to be, although significant, a poor marker.

CypC is the less known among the Cyps studied in the present work. It has been mostly described in the kidney, but it was also reported to be localized intracellular and extracellularly^19^. The physiological or pathological role of this protein is still unknown, although it was related to macrophages activation and the levels are increased in cerebral tissues after ischemia^29^. In our study, CypC levels were significantly increased in patients with acute CAD. In fact, the values were three times higher. Interestingly, this protein was significantly associated with CypA, CypB and CypD levels as well as with IL-6, a well-known inflammatory marker associated to the rupture of the atheroma plaque^5,30^. CypC has a significant role as CAD marker when values are up to 17.5 pg/mL with a high OR when other risk factors are also included. As far as we know, this is the first paper describing the values of CypC and its proposed usefulness as CAD marker. The capability of CypC to diagnose CAD presence was also showed through the ROC curve. The C-statistic value in this case was 0.89, meaning that if CypC values are high then there is a probability of 89% of developing CAD.

Moreover, traditional cardiovascular risk factors frequency (hypertension, dyslipidemia, smoking and age >50) is related to higher levels of CypA and CypC, whereas CypB levels are only related to hypertension and gender. The relationship of CypA and these parameters was already described, however this is the first study where human serum CypB and C levels are analyzed and related to CAD risk^21^. These results point again to the relevance of Cyps in CAD.

CypD has a key role regulating mitochondria function (opening the mPTP), mitochondria-dependent cell death, ischemia-reperfusion injury, and it has been also associated with atherosclerosis and diabetes^10,31^. The inhibition of CypD is postulated as a therapeutic strategy for cardio-protection in ischemia-reperfusion damage^31,32^. All these studies are based on the effect of CypD at mitochondrial level but no data about serum levels of this protein are available. In this paper we describe serum levels of CypD that although not significant, probably due to the population size, seems to be different depending or not on the presence or absence of the disease. Although the relevance of these data is unknown, it is worth mentioning that CypD can be quantified in serum levels and the profile in healthy subjects and patients is not the same. Further studies should be done to clarify these findings that cannot yet be explained.

The general link between Cyps levels and biochemical blood parameters and cells count require further specific studies to be understood. However, since many of them are significantly related to the presence of CAD, their correlation with CypA and CypC again reinforce the possible engagement of these inmunophilins in this process.

Several limitations should be considered in the present study. First the number of subjects involved in this study is relatively small, however it is representative of the number of cases of the health area. The same happens with gender and age of patients. It is well known that CAD diseases prevalence is higher in men and aging people^2^. However, to avoid bias in control subjects values a wide range of age in men and women were selected, although a further analysis should be done in a large population. Second, CypA plasma levels were before proposed as CAD biomarker and related with several pathological process^9,21^. In addition, CypB was recently related with metabolic syndrome^13^. However, CypC levels were not before studied. Third, total cholesterol and LDL values are higher in control subjects than in CAD patients due to antilipidemic treatments. Finally, DM was not considered as cardiovascular risk factor because of the limited number of control participants with this medical status, this will be considered in further studied. In addition, the intra and inter-assay coefficients of variation of the ELISA kits for Cyps determinations were <10%.

In summary, atherosclerosis is a vascular inflammatory disease, which involves several causative agents as oxidative stress among others. Migrating inflammatory cells produce large amounts of ROS and other pro-inflammatory stimuli that promote cardiovascular diseases. CypA has a key role in ROS production, as chemotactic agent and has been extensively associated to these diseases^14,19^. Since CypB and CypC are associated to redox regulation, their participation in those processes could be hypothesized.

Despite study limitations such as a study population relatively small, serum levels of CypC are clearly and significantly related with CAD. Further analysis in larger population would clarify the role of this protein. This paper describes for the first time that CypC levels are increased in CAD patients and confirms the role of CypA in this process. Among them, CypC serum levels highlight as a promising biomarker of CAD.

## Material and Methods

### Chemicals and solutions

Human Cyclophilin A ELISA kit (CSB-E09920H), Human Cyclophilin B ELISA kit (CSB-E11218H) and Human Cyclophilin C ELISA kit (CSB-EL018473HU) were obtained by Cusabio. Human Cyclophilin D ELISA kit (E-EL-H1936) was from Elabscience. Human High Sensitivity T Cell Magnetic Bead Panel Milliplex® map kit (#HSTCMAG-28SK; #HSTCMAG28SPMX13; #HSTCMAG28SOMX21; #HSTCMAG28PMX13BK and #HSTCMAG28PMX21BK) and Human MPP Magnetic Bead Panel 2 Milliplex® map kit (#HMMP2MAG-55K) were purchased from Merk (Madrid, Spain).

### Population study

An observational study about serum levels of Cyps was conducted in patients with symptoms of CAD referred to the Cardiology Department of Hospital Universitario Lucus Augusti in Lugo, Spain, from January 2017 to March 2018. A total of 40 patients with CAD were included. CAD disease was defined as prior myocardial infarction (MI), coronary revascularization or angiographic documentation of any significant coronary stenosis. CAD Patients with unstable angina suffering ischemic chess pain and/or electrocardiography abnormalities in the previous 24 hours were selected before receiving treatment. These patients were in the early onset of acute coronary syndrome. Coronary angiograms were evaluated by experienced cardiologists, who were blinded to the patients’ data. A narrowing of artery lumen by more than 51% of the diameter was considered as a clinically significant for CAD. Patients with chronic or acute inflammatory disease, rheumatic disease, cancer, autoimmune diseases, valvular disease or congestive heart failure were excluded. Also, 44 control subjects without known atherosclerosis disease, with normal angiography findings and normal serum cardiac biomarkers were enrolled in the study. All participants included in the present study belong to the public healthcare area of Lugo, Spain. The population to be studied was designed according to CAD prevalence in this area. The institutional and regional ethical board (Comité Autonómico de Ética da Investigación de Galicia, Comité Territorial de Ética da Investigación de Santiago-Lugo, Secretaria Xeral, Consellería de Sanidade, Xunta de Galicia) approved the study (Reference: 2016/508, Approved date: December 19, 2016, according to the principles outlined in the Declaration of Helsinki). Participants in the present study were informed and voluntarily written informed consent was obtained from all of them.

### Baseline measurements

Information on general vital status and significant clinical data were obtained for all the patients and control volunteers from their medical history and personal questionnaires. CAD as well as familiar CAD antecedents were recorded. Hypertension (HTA), dyslipidemia (DL), smoking status, age and gender were assessed as cardiovascular risk factors. The lipid-lowering drugs, hypoglycemic drugs and antiagregants consumption was also taken into consideration. HTA was considered when blood pressure was ≥140/90 mmHg. Diabetes mellitus (DM) was considered when fasting glucose levels were ≥126 mg/dl or if hypoglicemic treatment or insulin was used. DL was considered if low-density lipoprotein (LDL) cholesterol level was ≥140 mg/dl or high-density lipoprotein (HDL) cholesterol level was ≤40 mg/dl, or if an antilipidemic treatment was used.

### Blood sampling protocol

Peripheral blood was obtained from each participant from the antecubital vein using anti-coagulant free tubes (BD vacutainer serum tubes), in the first 24 hours after admission. The blood samples were allowed to clot for 20 min at room temperature before centrifugation (3,000 rpm for 10 minutes at 4 °C). Serum supernatants were collected and stored at −80 °C until needed for analysis. Samples were thawed once.

### Measurement of Cyclophilin A, B, C and D serum levels

Levels of serum CypA, CypB, CypC and CypD were measured using ELISA regarding manufacturer’s instructions. Before carrying out the experiments, serum samples were brought to room temperature, vortexed and centrifuged. Absorbance measurements were done in a microplate reader at 450 and 540 nm (only at 450 nm in the case of Cyp D). To correct optical imperfections, readings at 540 nm were subtracted from readings at 450 nm. All samples were run by duplicate and the concentrations were calculated using a standard curve. The range of determination was 3.12–200 ng/mL for CypA; 31.25–2,000 pg/mL for CypB; 23.5–1,500 pg/mL for CypC and 62.5–4,000 pg/mL for Cyp D. Serum levels below the lower limit of determination were undetectable and were considered as 0 pg/mL for statistical analysis. The intra and inter-assay coefficients of variation of the ELISA kits were <10%. No cross reactivity was observed between Cyp antibodies.

### Measurement of cytokines and metalloproteinases levels

Levels of serum interferon ϒ (IFNϒ), interleukin (IL)-1β, IL-2, IL-6, IL-8, tumor necrosis factor α (TNFα), metalloproteinase (MMP)-2 and MMP-9 were determined using a magnetic bead-based multiplex immunoassay (Milliplex® Map Kit) according to manufacturer’s instructions. Briefly, the serum was collected and kept at −80 °C until the time of performing the ELISA. Before the experiment, the samples were thawed, mixed by vortexing and centrifuged to remove particles. Serum samples were diluted (twenty times in the case of cytokines and twice in the case of MMPs) with buffer. First, the 96-well filter bottom well was pre-washed. 25 μL of each standard, control or samples were added to the appropriate wells with 25 μL of assay buffer provided in the kit and 25 µL of the mixed beads to each well. After that, the plate was incubated for 2 hours at room temperature, in the case of cytokines, and for 18 hours at 4 °C in the case of MMPs and washed three times with washing buffer. Thereafter, detection antibodies were added to each well and incubated for 1 hour at room temperature. The plate was washed as described before and Streptavidin-Phycoerythrin added to each well and incubated for 30 min at room temperature. All incubations were performed in an orbital shaker at 600 rpm. Finally, the plate was washed, and the median relative fluorescence units were recorded using a Luminex® 200™ with xPONENT® software. All samples were run by duplicate and the concentrations were calculated using a standard curve. The range of determination was 0.61–2,500 pg/mL for IFNϒ; 0.49–2,000 pg/mL for IL-1β and IL-2; 0.18–750 pg/mL for IL-6; 0.31–1,250 pg/mL for IL-8; 0.43–1,750 pg/mL for TNFα; 68–50,000 pg/mL for MMP-2 and 14–10,000 for MMP-9. Serum levels below the lowest limit of determination were undetectable and were considered as 0 pg/mL for statistical analysis. There was no or negligible cross-reactivity between the antibodies and any of the other analytes. The intra-assay coefficient of variation of the ELISA kits were <5%. The inter-assay coefficient of variation of the ELISA kits were <15% (in the case of IL-1β, IL-2, IL-8, TNFα and MMP-9) and <20% (in the case of IFNϒ, IL-6 and MMP-2).

### Statistical analysis

SPSS24 for Windows OS was used for the statistical analysis. Significance was set at p < 0.05. Participants were divided in two groups based on CAD diagnosis. Categorical variables were presented as percentages and continuous variables were presented as means ± SEM. Kolmogorov-Smirnov (with Lilliefors correction) was first performed as normality test. Those continuous variables with normal distribution were compared using a Student’s T test (including Levene’s test to assess the equality of variance), otherwise the non-parametric Mann-Whitney U test was used. In this way, basic characteristics, biochemistry parameters, and levels of Cyps and inflammation markers were compared. Categorical variables were compared using *Χ*^2^ test. Correlations (bivariate analysis) between Cyps and inflammation markers and blood parameters were analyzed using the Spearman rank correlation coefficient. Receiver-operating-characteristic curves (ROC) were constructed to assess the sensitivity and specificity of Cyps measurements to know the ability to diagnose CAD by means of the area under the curve (AUC) ROC and expressed as C-statistic^33,34^. In addition, CAD curves were used to obtain cut-off points for logistic regression. Specificity, sensitivity, positive predictive value (VPP), negative predictive value (VPN) and statistic power (P) for the risk score were also calculated. Subsequently, single logistic regression model (univariable and multivariable analysis) was used to evaluate the association between serum Cyps levels and traditional risk factors (gender, age, smoke, DL and HTA). Data were present following STROBE reporting guidelines.

## Data Availability

The datasets generated during and/or analysed during the current study are available from the corresponding author on reasonable request.
